# A Modular Plug-And-Play Sensor System for Urban Air Pollution Monitoring: Design, Implementation and Evaluation

**DOI:** 10.3390/s18010007

**Published:** 2017-12-22

**Authors:** Wei-Ying Yi, Kwong-Sak Leung, Yee Leung

**Affiliations:** 1 Institute of Future Cities, The Chinese University of Hong Kong, Shatin, N.T., Hong Kong 999077, China; wyyi1991@gmail.com (W.Y.Y); yeeleung@cuhk.edu.hk (Y.L); 2 Department of Computer Science and Engineering, The Chinese University of Hong Kong, Shatin, N.T., Hong Kong 999077, China; 3 Department of Geography and Resource Management, The Chinese University of Hong Kong, Shatin, N.T., Hong Kong 999077, China

**Keywords:** air pollution monitoring, wireless sensor network, modular sensor system, plug-and-play

## Abstract

Urban air pollution has caused public concern globally because it seriously affects human life. Modern monitoring systems providing pollution information with high spatio-temporal resolution have been developed to identify personal exposures. However, these systems’ hardware specifications and configurations are usually fixed according to the applications. They can be inconvenient to maintain, and difficult to reconfigure and expand with respect to sensing capabilities. This paper aims at tackling these issues by adopting the proposed Modular Sensor System (MSS) architecture and Universal Sensor Interface (USI), and modular design in a sensor node. A compact MSS sensor node is implemented and evaluated. It has expandable sensor modules with plug-and-play feature and supports multiple Wireless Sensor Networks (WSNs). Evaluation results show that MSS sensor nodes can easily fit in different scenarios, adapt to reconfigurations dynamically, and detect low concentration air pollution with high energy efficiency and good data accuracy. We anticipate that the efforts on system maintenance, adaptation, and evolution can be significantly reduced when deploying the system in the field.

## 1. Introduction

Urban air pollution has caused public concern worldwide because it seriously affects human life, including our health, living environment, and economy. According to the World Health Organization’s report in 2012, 7 million premature deaths worldwide were related to air pollution [1]. Environmental issues like global warming, acid rain, and haze are also caused by air pollution. Moreover, expenditure on public health is increasing rapidly due to excessive levels of air pollutants [2].

In order to mitigate these issues, conventional monitoring systems have been installed in urban areas. These systems provide authorized information to the decision makers and public to enhance and manage the urban environment. However, they are suffering from the extremely sparse spatio-temporal resolution [3]. For example, there are only 16 monitoring stations in Hong Kong covering an area about 2700 square kilometers and the pollution information is updated hourly [4]. Air pollution information with such low spatio-temporal resolution is inadequate for monitoring personal and acute exposures to air pollutants, which are proven to be critical to human health [5,6,7,8].

Thanks to the advancing technology, in the next generation air monitoring systems [9] with a great number of stationary, wearable, and/or vehicular sensor nodes deployed in the field, micro-level air pollution information and personal exposure warnings can be achieved promptly. However, the flexibilities of maintenance, reconfiguration, and deployment of the state-of-the-art systems are limited.

**Issue 1:** Recent studies mainly focus on the system architecture and implementation, data quality, energy efficiency, and networking techniques [10,11,12,13,14,15]. None of them have dealt with the practical issues in real-life deployment scenarios when thousands of sensor nodes will be deployed in the field. For example, to ensure optimum performances, maintenances on sensor nodes such as calibrating sensors, replacing components, etc., which are time-consuming and labor-intensive, are not negligible. Systems that are easy to maintain can dramatically reduce these costs.

**Issue 2:** The hardware configurations of the existing systems with real-time fine-grained air pollution information are usually fixed according to the applications (i.e., without any flexible modularity feature) [16,17,18,19,20,21]. It is extraordinary difficult or even impossible to modify or expand their sensing capabilities, or perform system upgrade and evolution. Typically, wearable sensor nodes in participatory sensing scenario [17] are carried by users who lack the background knowledge to deal with the sensor nodes. Sensor nodes that are easy to use and self-adaptable to reconfigurations are able to relax users’ burden.

In this paper, a Modular Sensor System (MSS) architecture and a Universal Sensor Interface (USI) are proposed to tackle these issues. An earlier version of this paper was presented at the IEEE Sensors 2016 Conference and was published in its proceedings [22]. This paper extends the preliminary work by (1) adding the literature review and the design goals of the proposed system in Section 2 and Section 3.1 respectively; (2) detailing the implementation of the proposed system in Section 3.3; and (3) enriching the Section 4 including comparisons with more similar systems, power consumption analysis, and calibrations of the sensor modules. The major contributions of this paper are:

- A Modular Sensor System (MSS) architecture with multiple WSN compatibility, configurable and expandable sensing capabilities, and plug-and-play ability;
- A Universal Sensor Interface (USI) enabling modular hardware/software design, configurable and expandable sensing capabilities, and plug-and-play ability;
- Implementing and evaluating a MSS sensor node prototype having six plug-and-play sensor modules (The sensor node supports at most 16 sensor modules logically and electronically. Limited by the size and weight, only six physical USI sockets were implemented. The number of the physical USI sockets can be easily upgraded to 16.) and supporting multiple WSNs, which has good applicability and can fit in different deployment scenarios.

The remainder of this paper is organized as follows. A literature review on the existing systems is performed in Section 2. In Section 3, the detailed design and implementation of the MSS architecture and USI are presented. A sensor node prototype adopting the MSS architecture and USI is evaluated and calibrated in Section 4. Section 5 presents the conclusions of this paper.

## 2. Related Works

Previous research has successfully constructed monitoring systems that provide real-time micro-level air pollution information, flexible data access, and user-friendly data visualization by utilizing the low-cost sensors and WSNs together with mobile and/or web apps.

Systems proposed in [12,14,16,17,20] were mainly focused on the system architecture and implementation. An urban scale wireless networking testbed was proposed and implemented in [16] based on 100 Wi-Fi enabled Linux PCs equipped with a set of low-cost sensors. In [20], a real-time air monitoring system was proposed and implemented by utilizing the low-cost sensors and the General Packet Radio Service (GPRS) wireless link. The sensor nodes in [16] were mounted on the streetlight poles and powered by electrical grid, while the sensor nodes in [20] were stationary and powered by solar panels. A vehicular sensor network based air monitoring system was proposed in [12]. In this system, the sensor nodes equipped with low-cost sensors and ZigBee wireless links were deployed on the public transportations that enlarged the geographical coverage of each sensor node. Different from the systems presented above, the sensor systems proposed in [14,17] aimed at enabling citizens to monitor their exposures to air pollution and simultaneously provide pollution information for further usages like political decision-making or building urban air quality maps. In [17], a handheld sensor node with six environmental sensors was implemented. This sensor node was able to transmit data tagged with Global Positioning System (GPS) information to user’s smartphone through Bluetooth or directly upload the data to a server through GPRS or 802.15.4 radio. In [14], a maker friendly sensor node with configurable sensors and a Bluetooth link was implemented. In this system, an NoSQL database was chosen for the backend server considering the need for storing the time sensory data at any time from many sources. All of these systems are able to provide fine-grained air pollution information through mobile and/or web apps in real-time.

In addition to the system architecture and implementation, there exist studies that put more attention on the data quality, energy efficiency, and networking techniques of the air monitoring systems. A neural network structure was proposed and implemented in [10] to improve the data quality of the acquired pollution data by considering the relationship between the pollution data and the ambient conditions. The GasMobile [19] system achieved high data accuracy by calibrating the low-cost sensors from time to time with the collocated conventional monitoring stations. Three novel techniques (i.e., a temporal n-gram augmented Bayesian room localization method; an air exchange rate based indoor air quality sensing method; and a zone-based proximity detection method for collaborative sensing) were proposed and implemented to improve the data accuracy and energy efficiency of the Mobile Air Quality Sensing (MAQS) [18] system. The energy consumption of the system proposed in [11] was optimized on sensor level, node level, and network level by performing dynamic gas sampling, people presence sensing, and nodes cooperating, respectively. A Clustering Protocol of Air Sensor (CPAS) network was proposed in [13] to improve the energy efficiency, life-time, and data rate of the network. In [21], a next generation air monitoring system was developed and evaluated with both laboratory and field tests. In order to correct the impacts of ambient conditions (i.e., temperature and relative humidity) on the electrochemical sensors, a multi-parameter correction algorithm was adopted. For the monitoring system proposed in [15], a Low Power Wide Area Network (LPWAN) was utilized to enlarge the network coverage to meet the communication requirements of a massive number of air quality sensors over a large sensing area. Adaptive duty cycle technique was employed in the sensor nodes for energy saving and hence enlarged the network lifetime.

Issues and challenges like privacy problem, user behavior, and data visualization were studied in [23,24,25]. In addition, a detailed implementation, including both hardware and software, of adding the electrochemical sensors to the OpenSense [26] box was presented in [27].

## 3. System Architecture and Implementation

In this section, the objectives of the Modular Sensor System (MSS) architecture are first described. Then, the detailed design of the MSS architecture and the Universal Sensor Interface (USI) are presented. A sensor node prototype adopting the MSS architecture and USI is also implemented in this section.

### 3.1. Design Goals

G.1 *Portability:* Compared to a stationary sensor node, a portable one enlarges its geographic coverage, which leads to higher spatial resolution of the data acquired [12,28], when it is carried by an active user, the public transportation, or even an Unmanned Aerial Vehicle (UAV) [3].
G.2 *Energy Efficiency:* The portability of a sensor node is achieved at the expense of limited size, weight, and power. A sensor node with high energy efficiency can increase the flexibility of maintenance by reducing the charging frequency.
G.3 *Multiple WSN Compatibility:* A sensor node with multiple WSN compatibility can be deployed in stationary, wearable, or vehicular scenarios by replacing the wireless module only, while having satisfying trade-off among network connectivity, energy consumption, and cost efficiency.
G.4 *Multiple Sensing Ability:* The multiple sensing ability enables performing data correction, compensation, and fusion on sensors’ readings [10,29,30]. Moreover, a sensor node with multiple sensors has advantages over that with single sensor in terms of energy and cost efficiency.
G.5 *Configurable and Adaptable Sensing Capability:* By employing modular software/hardware design, the number and types of sensors on a sensor node are user-configurable and self-adaptable (i.e., plug-and-play) for different kinds of monitoring scenarios that increase the system flexibility and usability, while the sensors’ sensing intervals are self-configured for power managing and detection maximizing purposes. System evolution is cost-efficiently achieved by developing advanced sensor modules for existing sensor nodes.

### 3.2. System Architecture Design

**Figure 1:**
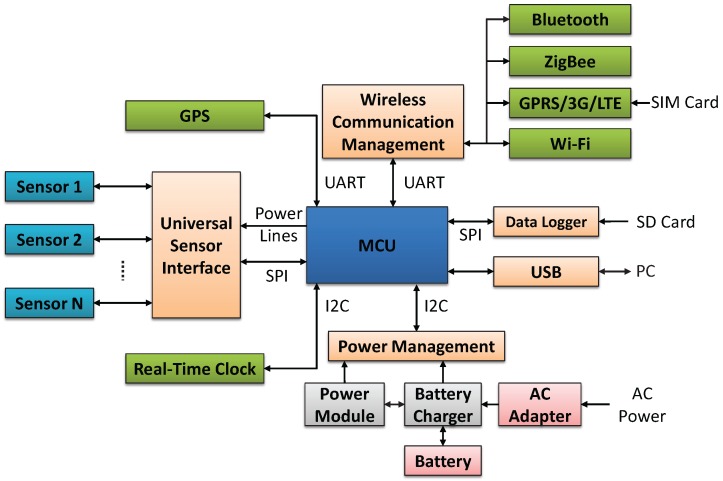
System architecture of a MSS sensor node. (GPS: Global Positioning System; SPI: Serial Peripheral Interface; UART: Universal Asynchronous Receiver/Transmitter; MCU: micro-controller unit; I2C: Inter-Integrated Circuit; GPRS: General Packet Radio Service; 3G: 3rd-Generation; LTE: Long Term Evolution; SIM: Subscriber Identification Module; SD: Secure Digital; USB: Universal Serial Bus; PC: Personal Computer; AC: Alternating Current.)

The system architecture design of the proposed MSS is illustrated in Figure 1 and the functionality of each block is presented as follows.

All of the sensors are connected to the micro-controller unit (MCU) through the proposed USI that enables the multiple sensing ability and the configurable and adaptable sensing capability described in G.4 and G.5, respectively. In addition, a lightweight Serial Peripheral Interface (SPI) based protocol is adopted in the USI, which is essential to the configurable and adaptable sensing capability described in G.5, to bridge the communication between the sensors and the MCU. The power lines of each sensor are controlled by the MCU for power managing (i.e., dealing with the energy efficiency described in G.2) and detection maximizing (e.g., configure the sensing interval of each sensor), and fault tolerance (e.g., restart or power down malfunction sensor) purposes.

The location and time information that tagged to the sensing data are acquired from a GPS module and a real-time clock module, respectively. The GPS module periodically updates the location information to the MCU through the Universal Asynchronous Receiver/Transmitter (UART) port and the time information is acquired from the real-time clock module by the MCU through the Inter-Integrated Circuit (I^2^C) port. Time information is also available from the GPS module and it is utilized to synchronize the real-time clock module when needed.

For the design goal described in G.3, multiple wireless communication modules (Bluetooth, ZigBee, Wi-Fi, GPRS, etc.) are supported by the wireless communication management block using the UART port. A sensor node is ready for a specific kind of deployment scenario (stationary, wearable, or vehicular) with satisfying network connectivity, energy consumption, and cost efficiency by replacing the wireless communication module with a proper one. For example, a Bluetooth module that has low energy consumption and price is used for a wearable sensor node to utilize a user’s smartphone that has promising network connectivity for uploading data to the central server.

A data logger using the SPI port is utilized to buffer the sensing data when the connection between the sensor node and the central server is not available. A Universal Serial Bus (USB) port is also available for development purpose. Typically, a sensor node is powered by battery and a battery power system using I^2^C port is adopted.

### 3.3. Detailed Implementation

A sensor node prototype adopting the MSS architecture and USI is implemented in this section. It consists of one Main-Body subsystem and multiple Sensor-Module subsystems.

#### 3.3.1. Main Body Subsystem

**Figure 2:**
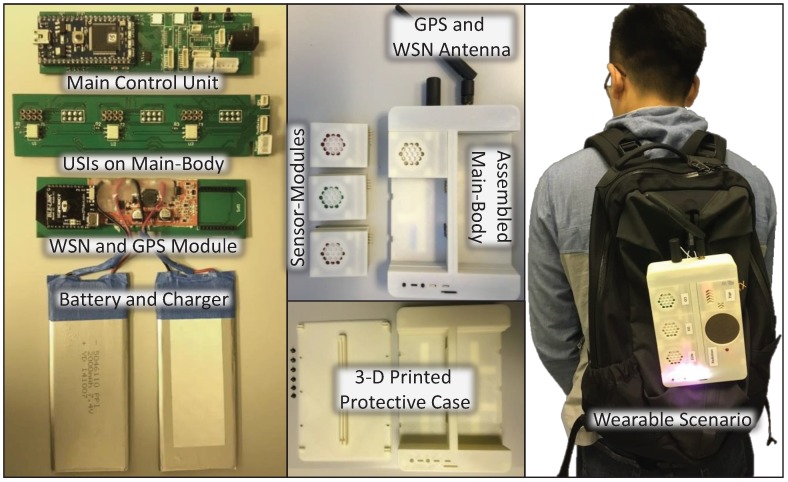
Main-Body subsystem and sensor nodes in wearable scenarios.

As illustrated in Figure 2, a Main-Body consists of a main control unit, six physical USI sockets, a WSN and GPS module (In wearable scenario where user carries the sensor node connected to the user’s smart phone, the GPS module is disabled and the location information from the user’s smart phone will be used instead.), two batteries with charging system, and a 3D printed protective case. It is 180.6 mm long by 122.6 mm wide by 36.0 mm high and weighs about 320.0 g.

**Figure 3:**
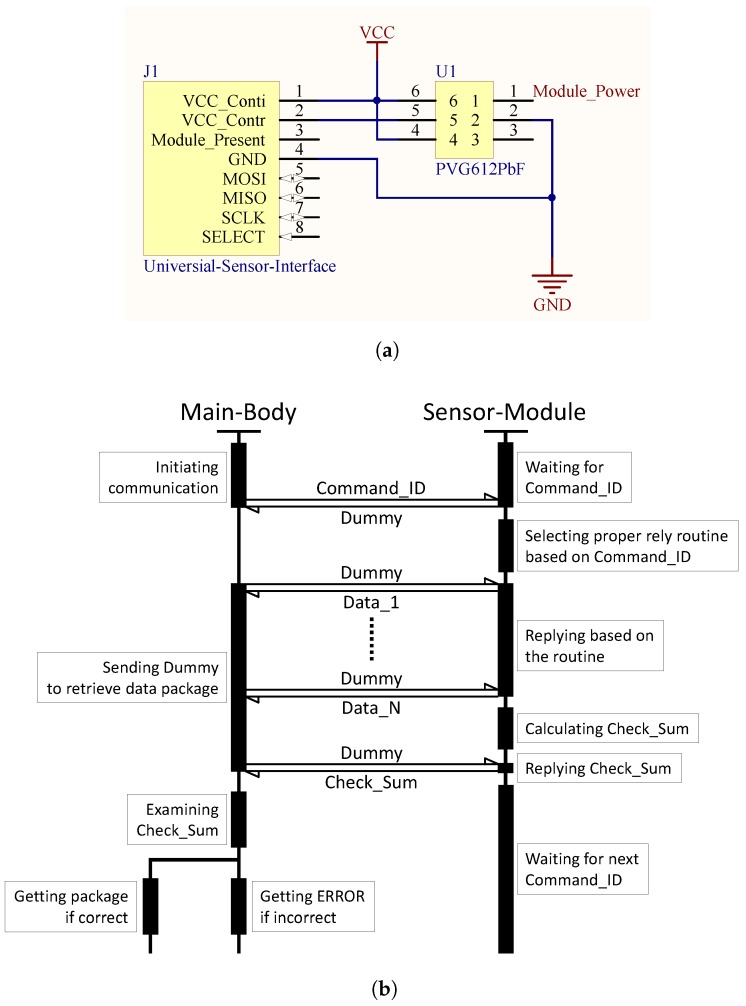
Schematic and communication protocol of the USI. (**a**) schematic of a USI on Main-Body; (**b**) timing diagram of the SPI based communication protocol.

An ARMmbed [31] LPC1768 platform is utilized in the main control unit. A real-time clock that keeps track of the current time is used and it communicates with the MCU through the I^2^C port. A micro Secure Digital (microSD) card based data logger for data buffering communicates with the MCU through the SPI port. To achieve the multiple sensing ability described in G.4, three 16-bit I^2^C input/output expanders are used to bridge the signals between the MCU and the pins *Module_Power*, *SELECT* and *Module_Present* of each USI illustrated in Figure 3a. Therefore, a main control unit is able to support up to sixteen USIs.

The schematic of a USI on the Main-Body is shown in Figure 3a. J1 is the USI’s 8-pin connector. Pin *VCC_Conti* powers the components that require continuous power in Sensor-Module. Controlled by the main control unit using the pin *Module_Power* on the solid-state relay U1, pin *VCC_Contr* powers the module control unit and other electronic components in a Sensor-Module for power managing, detection maximizing, and fault tolerating purposes mentioned in Section 3.2. Whenever a Sensor-Module is inserted into the Main-Body, pin *Module_Present* will be driven low and the main control unit is notified. It enables the adaptivity part (i.e., plug-and-play) of the configurable and adaptable sensing capability described in G.5. Pins *MOSI*, *MISO*, *SCLK*, and *SELECT* are standard SPI pins. The Main-Body communicates with the Sensor-Modules using a lightweight SPI based protocol and its timing diagram is presented in Figure 3b.

For the WSN and GPS module, because the Xbee [32] footprint is adopted, any Xbee packaged wireless modules can be applied in the Main-Body. The hardware compatibility of the multiple WSNs compatibility mentioned in G.3 is achieved. Multiple wireless technologies, including Bluetooth2.0/4.0, ZigBee PRO 2007, and IEEE802.11b/g/n, have been tested in the proposed system.

#### 3.3.2. Sensor-Module Subsystem

**Figure 4:**
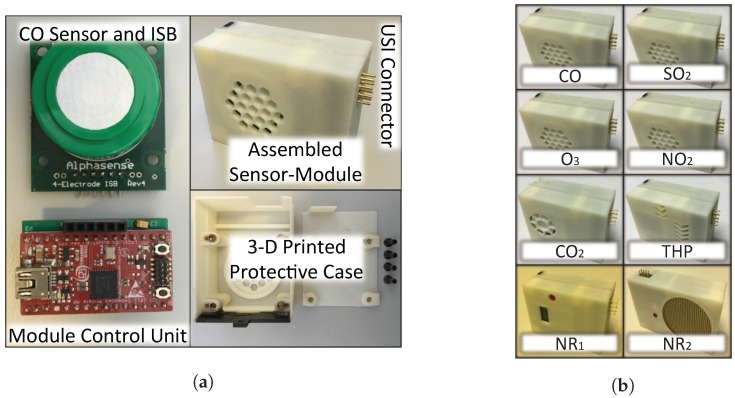
Sensor-Module subsystems. (**a**) a CO Sensor-Module subsystem; (**b**) eight implemented Sensor-Modules.

For a Sensor-Module, it consists of one or more sensors (depends on the sensors’ physical constraints like size, weight, power, etc.) with supporting circuits, a module control unit, a USI connector (J1 in Figure 3a), and a 3D printed protective case. A CO Sensor-Module illustrated in Figure 4a is presented as an example. It is 56.1 mm long by 44.0 mm wide by 26.0 mm high and weights about 45.0 g. In Figure 4b, eight types of Sensor-Modules have been implemented, namely CO, SO_2_, O_3_, NO_2_, CO_2_, THP, NR_1_, and NR_2_, and they are able to monitor the carbon monoxide, sulfur dioxide, ozone, nitrogen dioxide, carbon dioxide, temperature-humidity-pressure condition, β-γ radiation, and α-β-γ radiation, respectively. More Sensor-Modules are under development. Due to page limitation, only the detailed implementation of the gas pollution (CO, SO_2_, O_3_, and NO_2_) Sensor-Modules are presented in this section.

Based on the conclusions of [29,33] and the authors review on existing pollution sensing technologies [3], the solid-state and electrochemical sensors are suitable candidates for detecting the CO, SO_2_, O_3_, and NO_2_ pollution gases in the proposed system. Because the solid-state gas sensors are usually with higher power consumption, worse sensitivity, and lower resolution compared to the electrochemical ones, the Alphasense B4 series electrochemical gas sensors with Individual Sensor Boards (ISB) are selected. According to their specifications [34,35,36,37,38] summarized in Table 1, data with low noise and ppb-level resolution are achievable while having mW-level power consumption.

To achieve ppb-level resolution, a 6V low-noise Direct Current (DC) power supply is required by these sensors. Because a 2S Li-Po battery (8.4 V) is utilized in the Main-Body, a low-dropout (LDO) voltage regulator with the lowest output noise at the expense of slightly higher power consumption compared to other solutions [27] is adopted. Finally, the LF60AB LDO is selected as it has the highest output accuracy (6 ± 0.06 V) and lowest noise (50 μV) among all available LDOs.

According to Table 1, the lowest output resolution of the ISB is ≤1.192 mV. Because the build-in Analog-to-Digital Converter (ADC) of the module control unit (ARMmbed [31] LPC11U35 MCU) does not meet this requirement, a 16-bit 4-channel ADC with internal Programmable Gain Amplifier (PGA) named ADS1115 is utilized. A voltage divider is also applied to shrink down the ISB’s output range (0 to ≈6 V) to the ADC’s operating range (0 to 4.096 V).

In a gas pollution Sensor-Module, the gas sensor and ISB are powered by the pin *VCC_Conti* continuously while the module control unit is powered up by the pin *VCC_Contr* in each sensing interval. This mechanism enables high energy efficiency and good data quality at the same time because these gas sensors with ISB that consume mW-level power require about two hours to stabilized every time after switching on (Assuming each sensing interval is $T=t_{s}+t_{a}+t_{i}$, where $t_{s}$ is the sensor’s stabilization time, $t_{a}$ is the data acquisition time, and $t_{i}$ is the idle time before next sensing interval, and the power consumption of the gas sensor, ISB, and module control unit is $P_{S}$, $P_{\mathrm{ISB}}$, and $P_{\mathrm{MCU}}$, respectively. In order to achieve same level of data quality, the total energy consumption of each sensing interval with or without adopting the mechanism above is $E_{\mathrm{wi}}=(P_{S}+P_{\mathrm{ISB}})\cdot T+P_{\mathrm{MCU}}\cdot t_{a}$ and $E_{\mathrm{wo}}=(P_{S}+P_{\mathrm{ISB}}+P_{\mathrm{MCU}})\cdot (t_{s}+t_{a}),$ respectively. Given $(P_{S}+P_{\mathrm{ISB}})\ll P_{\mathrm{MCU}}$, $E_{\mathrm{wi}}\geq E_{\mathrm{wo}}$ stands only when $t_{i}\gg t_{s}$. In this manuscript, T=5 s and $t_{s}\gg T$ that result in $E_{\mathrm{wi}}<E_{\mathrm{wo}}$.).

## 4. Evaluation and Calibration

In this section, a functional evaluation including comparison between the MSS and similar systems, data acquisition and visualization, and power consumption analysis was performed. Then, the THP, CO, NO_2_, and O_3_ Sensor-Modules were calibrated by comparing the collocated and time-synchronized data from them with that from equipment of authorized agencies like the Hong Kong Observatory (HKO) and the Environmental Protection Department (EPD). The collocation calibration sites of the sensors should be carefully selected to ensure that the ambient conditions are similar to the locations where they are being used. If possible, the pollution levels of the calibration site should cover the maximum and minimum levels of the locations where the sensors are being deployed. Calibration on a more frequent basis may help ensure the most accurate data.

### 4.1. Functional Evaluation

Firstly, comparison between the MSS and other similar systems were performed as shown in Table 2. Most of them (systems without *Configurable* in the *Sensor Number* column) did not adopt any modular design in their systems and hence the hardware sets of these sensor nodes were fixed once they were designed, not to mention the plug-and-play feature (i.e., dynamically and automatically recognizing the inserted sensors without users’ configuration). For the Waspmote Pro (Libelium Comunicaciones Distribuidas S.L., Zaragoza, Spain) [14] and UPOD [39] sensor systems, their limited configurable sensing capabilities are based on the sensors’ analog interfaces that have drawbacks such as (1) inconsistent analog interfaces for different sensors from different manufactures, and (2) inconsistent calibration parameters for sensors inserted in different sensor nodes. For the NODE+ (Variable Inc., Chattanooga, TN, USA) [40] system, the configurable sensing capability is based on the digital interfaces on the sensor modules, which is similar to the proposed MSS. However, it supports two sensor modules at most and Bluetooth only while the MSS supports 16 sensor modules at most and multiple wireless modules. To the best of our knowledge, the proposed MSS is the first air monitoring system with (1) modular design, (2) plug-and-play feature, and (3) multiple WSNs compatibility.

**Figure 5:**
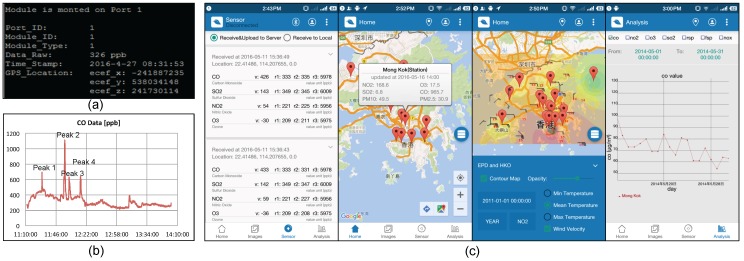
(**a**) raw data from the MSS sensor node through USB Port; (**b**) CO concentration over time (data were collected from 11:11:40 to 14:02:30 on 27 April 2016. The four labeled peaks accompanied the shuttle buses going by); (**c**) screenshots of the Android app (The first sub-figure shows data collected by the implemented MSS sensor node. The remaining sub-figures are other features of the app developed by the Institute of Future City of The Chinese University of Hong Kong, and the data displayed on them are from Hong Kong Observatory and Environmental Protection Department of Hong Kong.)

Secondly, as an illustrative example, a CO Sensor-Module was inserted into the Main-Body that was connecting to a PC through USB port for data acquisition. The inserted CO Sensor-Module was successfully identified and the data from it were handled automatically and properly as shown in Figure 5a. *Port_ID* is the inserted USI’s ID (from 1 to 16) and *Module_ID* is the Sensor-Module’s unique ID. *Module_Type* represents the type of this Sensor-Module and 1 means CO. *Data_Raw* is the current pollutant concentration in ppb level. *Time_Stamp* records the UTC+8 time at which the pollution information was acquired and *GPS_Location* in Earth Centered Earth Fixed (ECEF) mode is also tagged. Three hours of data as presented in Figure 5b were collected that the data acquisition rate was 5 s and the sensor node was installed next to the campus road. In this graph, four peaks are labeled and their appearances accompanied the shuttle buses going by. This indicates that the sensor node is able to capture micro-level air pollution information and in-time personal exposure warnings can be issued as appropriate. An Android app was also implemented. It utilizes Bluetooth to collect data from the MSS sensor node. The acquired data will then be uploaded to the central server using a cellular network or Wi-Fi. The screenshots of the app are shown in Figure 5c.

**Figure 6:**
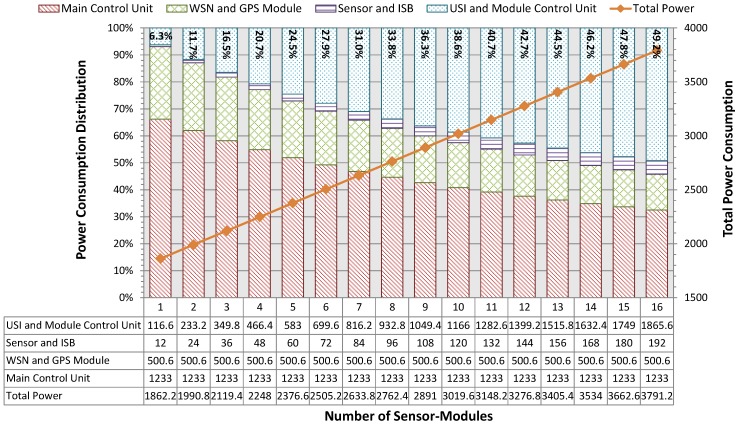
Power consumption distribution of the MSS sensor node with different numbers of gas Sensor-Modules (The data table is showing the exact power consumption of each major component with respect to the number of gas Sensor-Modules inserted. The unit of power consumption is mW. Each indicator in percentage on top of the rectangle column is the proportion of the total power consumption that was contributed by the USIs and module control units.)

Thirdly, a power consumption analysis of the implemented MSS sensor node was performed. The power consumption of each major component of the Main-Body and the gas pollution (CO, SO_2_, O_3_, and NO_2_) Sensor-Module is listed in Table 3. The LEDs and relays on the main control unit are used to indicate system status and control power supplies, respectively. The USI’s relay and the module control unit with supporting circuits (LDO voltage regulator, ADC, etc.) that highlighted in Table 3 are the essential components of the configurable and adaptable sensing capability described in G.5. Extra power is consumed by these highlighted components to achieve the desired features. As illustrated in Figure 6, although the power consumption of the highlighted components contributes only 6.3% to the total power consumption if 1 Sensor-Module is plugged in, it becomes more and more significant when the number of Sensor-Modules increases and 49.2% of the total power is consumed by them if 16 Sensor-Modules are inserted. Such issue is well considered when designing the MSS and USI. As mentioned in Section 3.2 and Section 3.3.2, these highlighted components of each Sensor-Module will only be switched on when data acquisition is required. Assuming the data acquisition process will take 0.1 s (in fact, less than 0.1 s) and the sensing interval is 5 s, only 0.13% and 1.9% of the total energy in one sensing interval is consumed by the highlighted components with 1 and 16 Sensor-Modules, respectively.

### 4.2. Collocation Calibration

**Figure 7:**
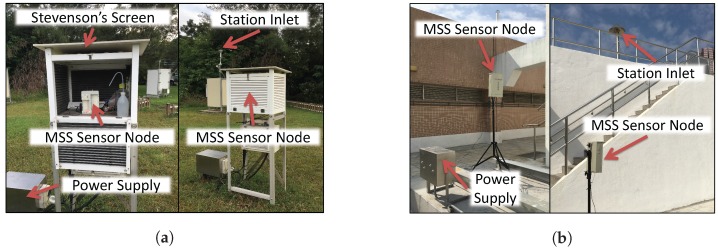
Collocation calibration sites. (**a**) HKO King’s park site; (**b**) EPD Tseung Kwan O site.

In this section, the THP, CO, NO_2_, and O_3_ Sensor-Modules were calibrated by comparing the collocated and time-synchronized data from them with that from equipment of authorized agencies like Hong Kong Observatory (HKO) and Environmental Protection Department (EPD) of Hong Kong. For the THP Sensor-Module, the sensor node was placed inside the Stevenson’s screen, which is next (≈5 meters) to the HKO’s monitoring station in King’s Park as shown in Figure 7a, and collected data consecutively for 6 days. For the CO, NO_2_, and O_3_ Sensor-Modules, the sensor node with a weatherproof case was placed next (≈4 meters) to the EPD’s monitoring station in Tseung Kwan O as shown in Figure 7b, and collected data consecutively for 23 days. Although the sensor node took a measurement every five seconds, we only have access to the 1-minute averaged data from HKO’s and EPD’s equipment. Therefore, we averaged the 12 measurements from the sensor node in each minute and used the averaged value to represent the measurement of that 1-minute interval.
**Algorithm 1:** Pairwise calibration algorithm proposed in [46]
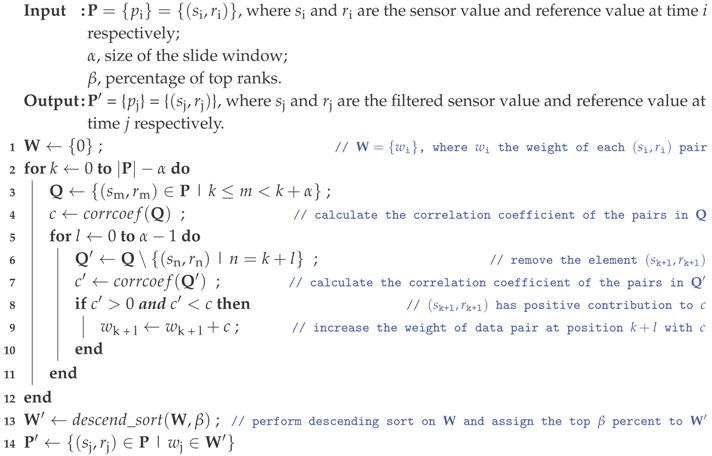


**Figure 8:**
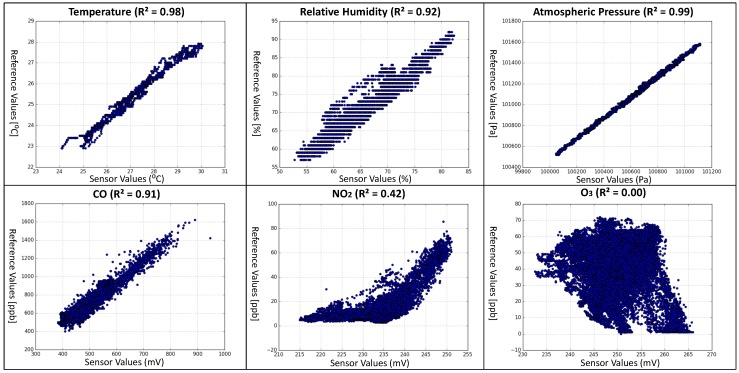
Scatter plots of sensor value (horizontal axises) and reference value (vertical axises) pairs of each monitoring species (The temperature data pairs were preprocessed using the filter proposed in [46] with a 6-h window and 20% top ranks. The $R^{2}$ is the square of the correlation coefficient.)

The key idea of collocation calibration is that if the sensor node and the reference station are collocated, they are likely observing the same phenomenon and the acquired data pairs should be highly correlated [46]. A relationship of these collocated and time-synchronized data pairs (In order to identify the events for which the sensor node and the reference station were monitoring the same phenomenon, a data filter proposed in [46] was adopted with a 6-h window and 20% top ranks. The filter algorithm can be found in Algorithm 1. Because this filter was evaluated using a temperature sensor in the original paper, only the temperature data pairs were filtered in our experiment.), which are plotted in Figure 8 with the horizontal axises representing the sensor values and the vertical axises representing the reference values, respectively, can be established by performing a polynomial regression (For the THP Sensor-Module and CO Sensor-Module, 1st order polynomial regression seems to be a good approximation because the improvement of using 2nd, 3rd, and 4th order polynomials is less than 2% in terms of mean squared error. For the NO_2_ Sensor-Module, 3rd order polynomial regression seems to be a good approximation because the improvement of using 1st, 2nd, and 4th order polynomials is −82%, −7%, and 3%, respectively, in terms of mean squared error.) on them. Calibrated sensor values, with respect to the reference values, are achieved by filling the sensor values to the calibration functions generated by the polynomial regression.

Throughout our discussion, we assume that the relationships of the sensor value and reference value pairs of each monitoring species are polynomial, and that the calibration functions are of the following format:

(1) $$\hat{p}\left(x_{i}\right)=a_{0}+a_{1}x_{i}+a_{2}{x_{i}}^{2}+\ldots +a_{n}{x_{i}}^{n},$$

where $x_{i}$ is the sensor value at time *i*, and $\hat{p}\left(x_{i}\right)$ is the calibrated sensor value, with respect to sensor value $x_{i}$ at time *i*, using calibration parameter set $\left\{a_{0},a_{1},a_{2},\ldots ,a_{n}\right\}$. We quantify the performances of the calibrated sensors using the *Mean Absolute Error (MAE)* and the *Standard Deviation (SD)*, which are defined as follows:

(2) $$MAE=\frac{1}{N}\sum_{i=1}^{N}\left|\hat{p}\left(x_{i}\right)-r\left(x_{i}\right)\right|,$$

(3) $$SD=\sqrt{\frac{1}{N}\sum_{i=1}^{N}\left(\right|\hat{p}\left(x_{i}\right)-r\left(x_{i}\right)|-MAE{)}^{2}},$$

where $r(x_{i})$ is the reference value corresponding to sensor value $x_{i}$ at time *i*, and *N* is the number of sensor value and reference value pairs of the monitoring species. In instrumentation and measurement society, the *MAE* is defined as *accuracy* and the *SD* represents the *precison*, *stability* or *repeatability* of a sensor.

In order to identify over-fitting, the data pairs of each monitoring species were randomly divided into two parts, namely training set (80%) and testing set (20%). The *Correlation Coefficients (CC)* for both training and testing sets were then calculated. The calibration parameter set of each monitoring species was achieved by performing polynomial regression on the training set. The calibration parameter set was then applied to the training and testing sets to find out the calibrated sensor value $\hat{p}\left(x_{i}\right)$ for each sensor value $x_{i}$ at time *i*. Finally, the *Mean Absolute Error (MAE)* and *Standard Deviation (SD)* of both training and testing sets were calculated using Equations (2) and (3), respectively. The procedures above were repeated five times and the results are listed in Table 4. We evaluate the regression performance of each monitoring species by looking at the mean difference between the training *MAE* and testing *MAE* (*MDMAE*), and the mean difference between the training *SD* and testing *SD* (*MDSD*) of all iterations that are defined as follows:

(4) $$MDMAE=\frac{100\%}{n}\sum_{i=1}^{n}\left(\frac{|MAE_{i}^{Test}-MAE_{i}^{Train}|}{MAE_{i}^{Train}}\right),$$

(5) $$MDSD=\frac{100\%}{n}\sum_{i=1}^{n}\left(\frac{|SD_{i}^{Test}-SD_{i}^{Train}|}{SD_{i}^{Train}}\right),$$

where $MAE_{i}^{Test}$, $MAE_{i}^{Train}$, $SD_{i}^{Test}$, and $SD_{i}^{Train}$ are the testing *MAE*, training *MAE*, testing *SD*, and training *SD* values in iteration *i*, respectively.

**Figure A1:**
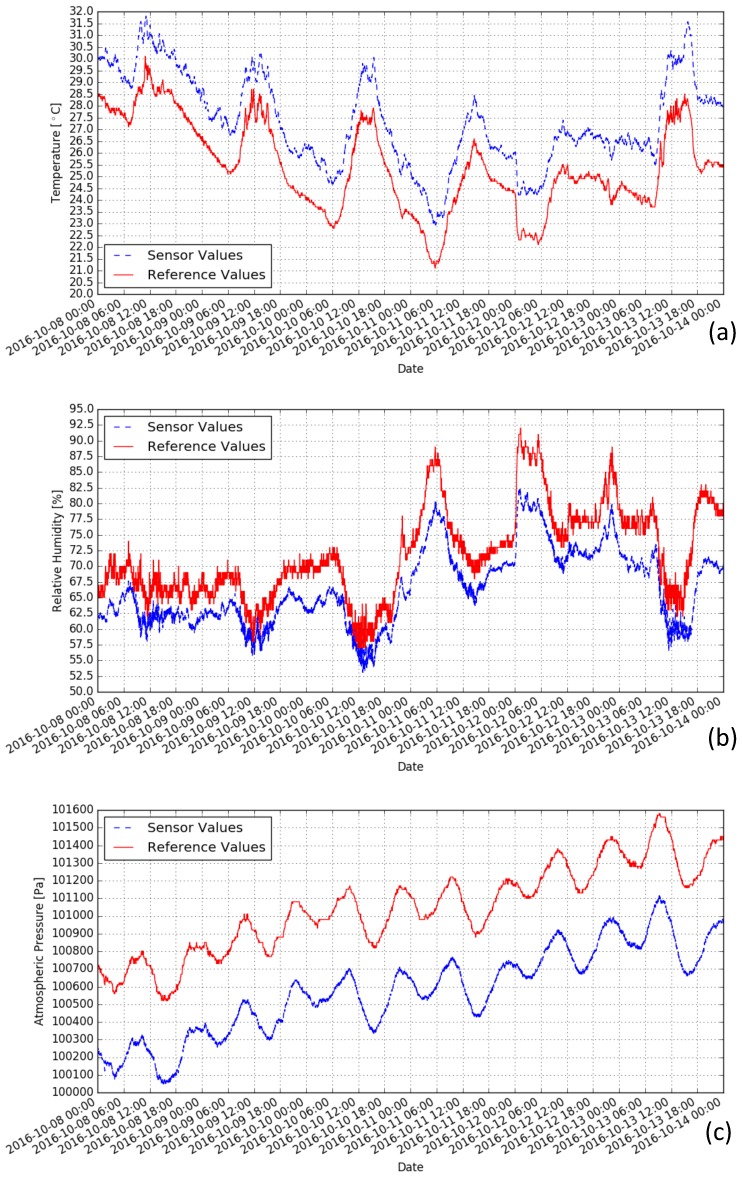
Line plots of sensor values from THP Sensor-Module and reference values from HKO’s station. (**a**) temperature data pairs; (**b**) relative humidity data pairs; (**c**) atmospheric pressure data pairs.

For the THP Sensor-Module, the SHT31 temperature-humidity sensor and the BMP280 barometer are utilized. The sensor node with the THP Sensor-Module was collocated with HKO’s monitoring station and the data acquired from the 6-day measurement campaign (over 8000 data pairs per species) are shown in Figure A1. The sensor value and reference value pairs of each monitoring species are highly correlated and the *squared correlation coefficients (R^2^)* are 0.98 (temperature), 0.92 (relative humidity), and 0.99 (atmospheric pressure), respectively. From the scatter plots of the sensor value and reference value pairs of temperature, relative humidity, and atmospheric pressure illustrated in Figure 8, they show clear linear relationships between the sensor values and reference values. Moreover, for each monitoring species, the sensor values and reference values have the same unit (i.e., °C, %, or Pa), and the relationships of the reference values and the differences between reference and sensor values are not significant (The *R*^2^ values of the reference values and the differences between reference and sensor values for temperature, relative humidity, and atmospheric pressure are 0.00, 0.41, and 0.03, respectively. Although the *R*^2^ value of relative humidity is significantly larger than that of temperature and atmospheric pressure due to the fact that the accuracy of typical humidity measuring equipment decreases when humidity level increases, we consider it as insignificant relationship for simplicity reason.). Therefore, the calibration parameter set $\left\{a_{0},a_{1},a_{2},\ldots ,a_{n}\right\}$ can be achieved by setting $a_{0}=\frac{1}{N}\sum_{i=1}^{N}\left(r\left(x_{i}\right)-x_{i}\right)$, $a_{1}=1.0$, and $a_{n}=0$ for n≥2, respectively.

For the CO, NO_2_, and O_3_ Sensor-Modules, the Alphasense CO-B4, NO_2_-B4, and O_3_-B4 sensors are utilized, respectively. The sensor node with these three Sensor-Modules was collocated with EPD’s monitoring station and the data acquired from the 23-day measurement campaign (over 33,000 data pairs per species) are shown in Figure A3. The sensor value and reference value pairs for CO are highly correlated with $R^{2}=0.91$. For the NO_2_ data pairs, although the *R*^2^ value drops down to 0.42, we still consider it as significant. For the O_3_ data pairs, the *R*^2^ value is close to 0. Such insignificant correlation may be caused by the fact that the O_3_ sensor reacts to both ozone and nitrogen dioxide [36]. Currently, we conclude that our calibration method is not suitable for this O_3_ sensor and the calibration of the O_3_ Sensor-Module is considered as future work. From the scatter plots of the sensor value and reference value pairs for CO and NO_2_ illustrated in Figure 8, the CO data pairs show a clear linear relationship and the NO_2_ data pairs shows a clear nonlinear relationship. Preliminary results indicated that the 1st order and the 3rd order polynomial regressions seem to be good approximations to the CO data pairs and NO_2_ data pairs, respectively.

Referring to the calibration results of the temperature, relative humidity, atmospheric pressure, CO, and NO_2_ data pairs listed in Table 4, the *MDMAEs* calculated using Equation (4) are 3.6%, 1.7%, 1.5%, 0.9%, and 1.1% respectively, and the *MDSDs* calculated using Equation (5) are 3.2%, 1.7%, 0.7%, 0.9%, and 1.8%, respectively. We consider that the *MDMAE* and *MDSD* values of all these five monitoring species are relatively small, which means the calibration parameter sets achieved from the training sets perform well in the testing sets. Hence, we conclude that no over-fitting is observed.

**Figure 9:**
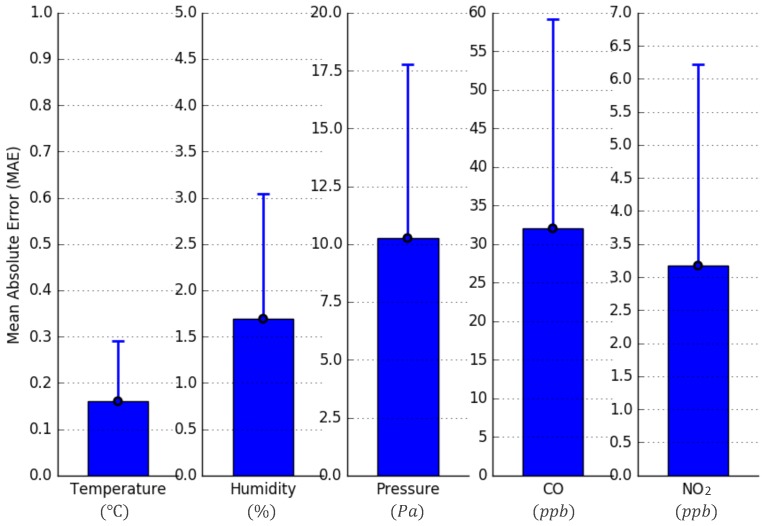
The Mean Absolute Error (MAE) and Standard Deviation (SD) values of the temperature, relative humidity, atmospheric pressure, CO, and NO_2_.

**Figure A2:**
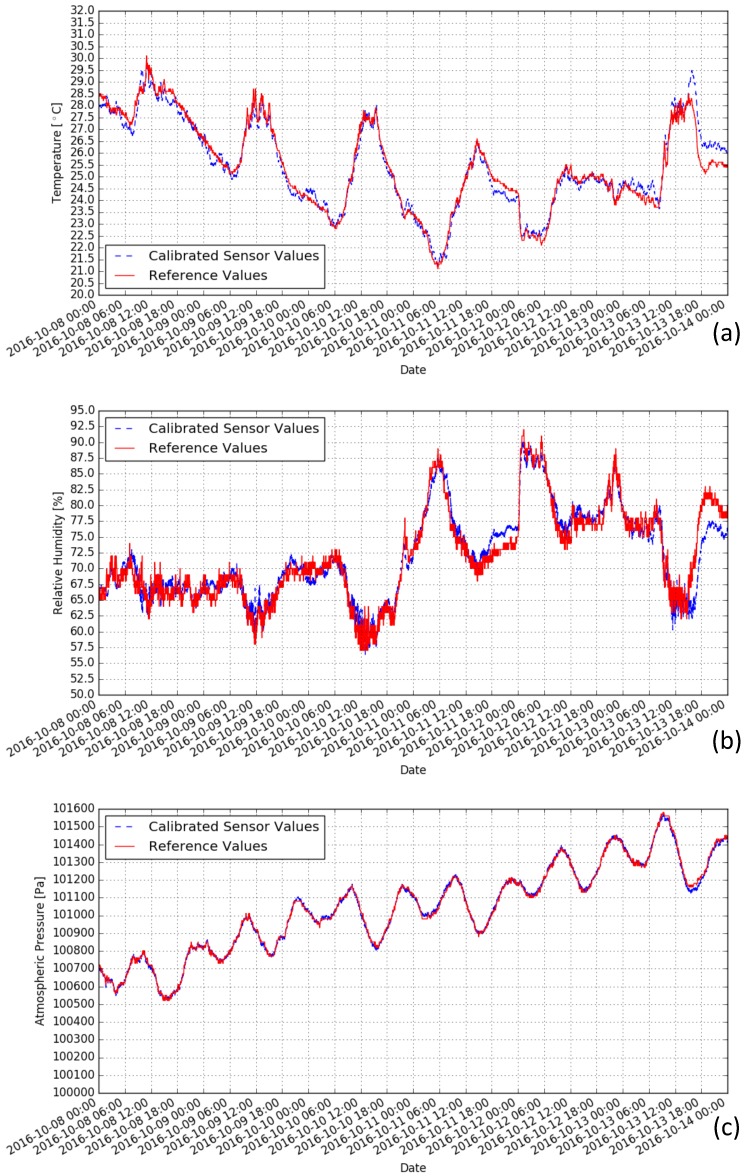
Line plots of calibrated sensor values from THP Sensor-Module and reference values from HKO’s station. (**a**) temperature data pairs; (**b**) relative humidity data pairs; (**c**) atmospheric pressure data pairs.

**Figure A3:**
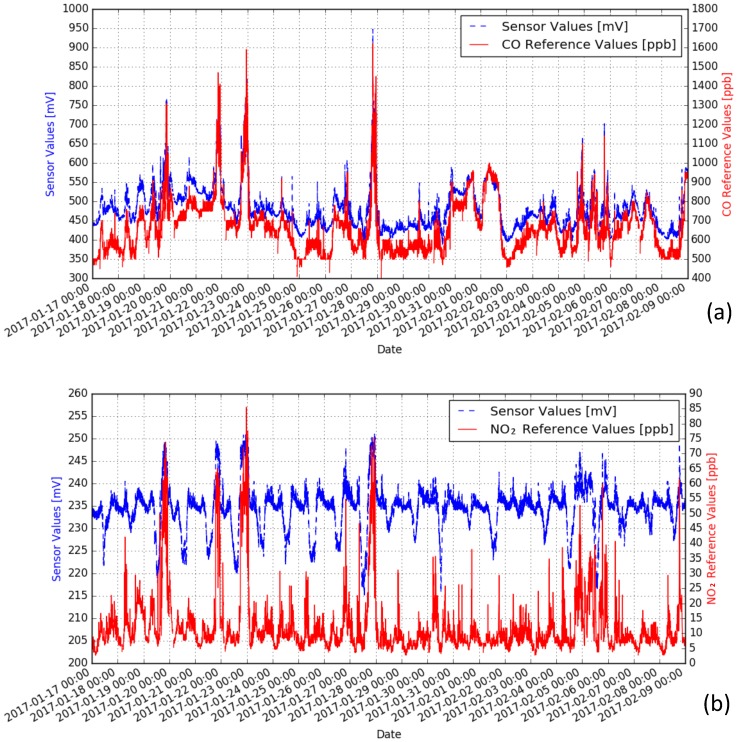
Line plots of sensor values from CO and NO_2_ Sensor-Modules, and reference values from EPD’s station. (**a**) CO data pairs; (**b**) NO_2_ data pairs.

**Figure A4:**
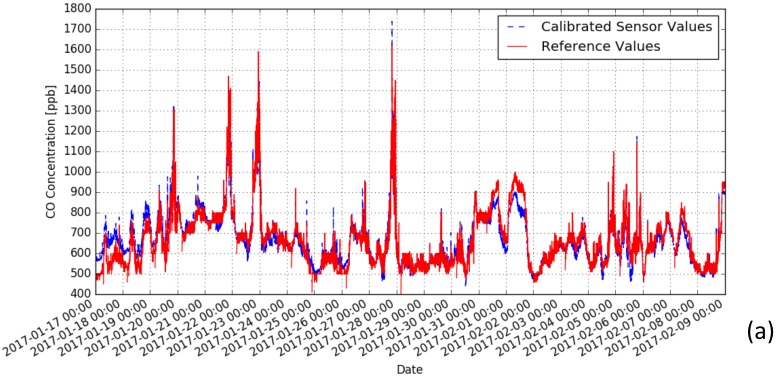

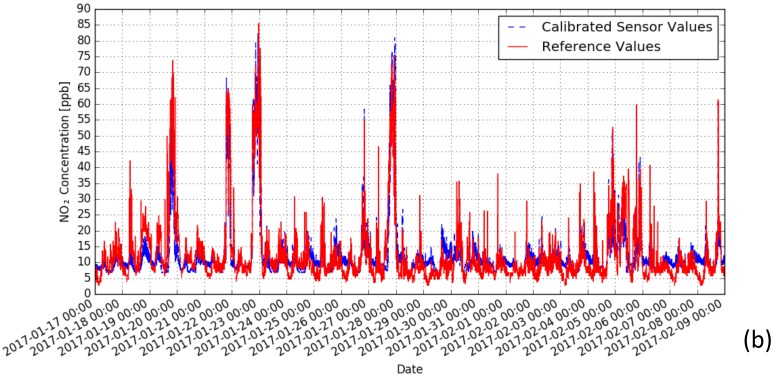
Line plots of calibrated sensor values from CO and NO_2_ Sensor-Modules, and reference values from EPD’s station. (**a**) CO data pairs; (**b**) NO_2_ data pairs.

Finally, the calibration parameter sets for the temperature, relative humidity, atmospheric pressure, CO, and NO_2_ sensors were calculated by performing polynomial regressions on all data pairs of each monitoring species. The calibrated sensor values and reference values are plotted in Figure A2 and Figure A4. The *MAE* and *SD* values of each monitoring species were then calculated and shown in Figure 9. For the temperature, relative humidity, and atmospheric pressure data pairs, the *MAE* values equal to 0.16, 1.69, and 10.26, respectively. This means that the accuracies of the calibrated temperature, relative humidity, and atmospheric pressure sensors, with respect to the HKO’s reference station, are $\pm 0.16{\;}^{\circ }$C, ±1.69%, and ±10.26 Pa, respectively. All these values are within the accuracy tolerances listed in the sensors’ specifications, which are $\pm 0.3{\;}^{\circ }$C, ±2%, and ±12 Pa, respectively. We conclude that the THP Sensor-Module is successfully calibrated. For the CO and NO_2_ data pairs, the *MAE* values are 32.03 and 3.12, respectively. Hence, the accuracies of the calibrated CO and NO_2_ sensors, with respect to EPD’s reference station, are ±32 ppb, and ±3 ppb, respectively. Considering that the maximum concentrations of the CO and NO_2_ from the 23-day measurement campaign are about 1700 ppb and 70 ppb, and that EPD [47] sets their objectives to ≤30,000 μg/m${}^{3}$ (equivalent to 26,200 ppb, 1-h average) and ≤200 μg/m${}^{3}$ (equivalent to 106 ppb, 1-h average), respectively, such accuracies are acceptable.

## 5. Conclusions

In this paper, we proposed the Modular Sensor System (MSS) architecture and the Universal Sensor Interface (USI) to address the fixed hardware configuration issue of existing air monitoring systems. We adopted the modular design into the proposed architecture, which enables it with multiple WSNs compatibility, multiple sensing capability, and configurable and adaptable sensing capability. We anticipate that the flexibilities of maintenance and usability of the monitoring system adopting the proposed architecture are improved compared to similar systems. To the best of our knowledge, the proposed MSS is the first air monitoring system with (1) modular design, (2) plug-and-play feature, and (3) multiple WSNs compatibility. In Section 3.3 and Section 4.1, a MSS sensor node prototype was implemented and evaluated. Evaluation results show that MSS sensor nodes can easily fit in different scenarios, adapt to reconfigurations dynamically, and detect low concentration air pollution with high energy efficiency and good data accuracy. In Section 4.2, the implemented THP, CO, and NO_2_ Sensor-Modules were calibrated by comparing the data from them with that from the reference equipment of authorized agencies. The calibrated accuracies of the temperature, relative humidity, atmospheric pressure, CO, and NO_2_ sensors are $\pm 0.16{\;}^{\circ }$C, ±1.69%, ±10.26 Pa, ±32 ppb, and ±3 ppb, respectively. All these values are within acceptable ranges and we concluded that the THP, CO, and NO_2_ Sensor-Modules were successfully calibrated. The implementation and calibration of more Sensor-Modules are considered as future work.

## Figures and Tables

**Table 1:** Technical detail of Alphasense sensors and Individual Sensor Boards (ISB).

Parameters	CO	NO_2_	SO_2_	O_3_	NO	H_2_S
Precision ^ [ppb]	4	15	5	15	15	1
Sensor Sensitivity ^ [nA/ppb]	0.525	−0.425	0.35	−0.4	0.65	1.75
ISB Gain ^ [mV/nA]	0.8	−0.726	0.8	−0.746	0.8	0.8
Working Electrode Sensitivity * [mV/ppb]	0.42	0.309	0.28	0.298	0.52	1.4
Working Electrode Zero Offset ^ [mV]	270	225	355	260	545	350
Auxiliary Electrode Zero Offset ^ [mV]	340	245	345	300	510	350
Output Resolution [mV]	1.68	3.708	1.4	1.192	7.8	1.4
Full Scale Range @ 6V [ppm]	13	18	20	19	10	4
Maximum Output [V]	5.46	5.562	5.6	5.662	5.2	5.6

* The Working Electrode Sensitivities are achieved using the ISB. ^ These are typical values and the actual values can be achieved by calibration.

**Table 2:** Comparison between the MSS and similar systems. (VOC: Volatile Organic Compound; PM: Particulate Matter; MAQS: Mobile Air Quality Sensing; GPRS: General Packet Radio Service; MAS: Mini Air Station; GSM: Global System for Mobile Communications; UMTS: Universal Mobile Telecommunications System; WLAN: Wireless Local Area Network; PID: Photo-Ionization Detector.)

System Name	Number of Sensors	Type of Sensor	Type of WSN	Deployment Scenario
Waspmote PRO [14]	Configurable (max. 5)	CO, NO_2_, O_3_, VOC, humidity, temperature	Bluetooth	Wearable, Vehicular
Monitoring Node [15]	3	PM_2.5_, humidity, temperature	802.15.4k	Stationary
CitySense [16]	5	temperature, humidity, pressure, etc.	Wi-Fi	Stationary
CommonSense [17]	6	CO, O_3_, NO_X_, temperature, humidity, etc.	Bluetooth, 802.15.4, GPRS	Wearable
MAQS [18]	4	CO_2_, light, humidity, etc.	Bluetooth	Wearable
GasMobile [19]	1	O_3_	None (cable)	Wearable
Multi-Gas Monitoring Stations [20]	3 or 5	O_3_, NO_2_, CO, etc.	GPRS	Stationary
MAS [21]	6	NO_2_, CO, O_3_, PM_2.5_, humidity, temperature	GSM	Stationary
OpenSense [41]	6	O_3_, CO, NO_2_, fine particles, temperature, humidity	GPRS/UMTS, WLAN	Stationary, Vehicular
UPOD [39]	Configurable (max. 13)	CO_2_, PID, humidity, temperature, metal-oxide sensor, etc.	Wi-Fi	Stationary, Vehicular
Speck [42]	1	PM_2.5_	Wi-Fi	Indoor
AirBeam [43]	1	PM_2.5_	Bluetooth	Wearable
AirQualityEgg [44]	7	NO_2_, CO, temperature, humidity, etc.	Wi-Fi	Indoor
AirBoxx Monitor [45]	8	CO, CO_2_, temperature, humidity, etc.	Bluetooth	Indoor
NODE+ [40]	Configurable (max. 2)	CO, CO_2_, H_2_S, NO, NO_2_, SO_2_, temperature, humidity, etc.	Bluetooth	Wearable
MSS	Configurabl (max. 16)	Configurable and Expandable	Bluetooth, GPRS, ZigBee, Wi-Fi, etc.	Stationary, Wearable, and Vehicular

**Table 3:** Power consumption of each major component on the MSS sensor node. (MCU: micro-controller unit; LED: Light Emitting Diode; WSN: Wireless Sensor Network; GPS: Global Positioning System; USI: Universal Sensor Interface; ISB: Individual Sensor Board.)

Component Name		Voltage (V)	Current (mA)	Power (mW)
Main Control Unit	MCU	8.4	51	428.4
	LED 1	8.4	36	302.4
	LED 2	3.3	18	59.4
	Relay 1	5	16	80.0
	Relay 2	5	16	80.0
	Remaining Circuits	-	-	282.8
WSN and GPS Module	Bluetooth	3.3	12	39.6
	GPS	3.3	47	155.1
	Remaining Circuits	-	-	305.9
USI	Relay	3.3	13	42.9
Gas Sensor-Module	Module Control Unit	5	7	35
	Sensor and ISB	6	2	12
	Remaining Circuits	-	-	38.7

**Table 4:** Calibration results of THP (temperature-humidity-pressure) Sensor-Module, CO Sensor-Module, and NO_2_ Sensor-Module.

Iteration			1	2	3	4	5
Temperature * ^‡^ (°C)	Training Set	CC	0.988	0.987	0.987	0.988	0.987
		MAE	0.160	0.163	0.162	0.162	0.163
		SD	0.133	0.130	0.132	0.132	0.131
	Testing Set	CC	0.987	0.987	0.988	0.987	0.989
		MAE	0.166	0.156	0.159	0.159	0.153
		SD	0.125	0.133	0.125	0.130	0.130
Relative Humidity ^‡^ (%)	Training Set	CC	0.962	0.961	0.962	0.962	0.962
		MAE	1.697	1.684	1.684	1.696	1.685
		SD	1.358	1.360	1.350	1.357	1.345
	Testing Set	CC	0.961	0.962	0.960	0.960	0.958
		MAE	1.673	1.714	1.718	1.671	1.716
		SD	1.352	1.355	1.391	1.360	1.407
Atmospheric Pressure ^‡^ (Pa)	Training Set	CC	0.999	0.999	0.999	0.999	0.999
		MAE	10.24	10.25	10.31	10.25	10.25
		SD	7.521	7.499	7.517	7.492	7.492
	Testing Set	CC	0.999	0.999	0.999	0.999	0.999
		MAE	10.41	10.40	10.13	10.38	10.39
		SD	7.460	7.516	7.466	7.569	7.544
CO ^(ppb)	Training Set	CC	0.952	0.952	0.952	0.953	0.951
		MAE	31.99	31.97	32.14	32.02	32.04
		SD	27.04	27.14	27.18	27.03	27.14
	Testing Set	CC	0.953	0.952	0.951	0.948	0.956
		MAE	32.35	32.44	31.84	32.29	32.14
		SD	27.50	27.12	26.88	27.52	27.15
NO_2_^†^ (ppb)	Training Set	CC	0.643	0.647	0.643	0.642	0.646
		MAE	3.180	3.190	3.166	3.169	3.180
		SD	3.031	3.056	3.024	3.038	3.030
	Testing Set	CC	0.660	0.644	0.643	0.642	0.648
		MAE	3.190	3.159	3.230	3.216	3.200
		SD	3.070	2.954	3.110	3.058	3.064

* The sensor value and reference value pairs were preprocessed using the filtered proposed in [46] with 6-h window and 20% top rank. ^‡^ Calibration parameter set is achieved by setting $a_{0}=\frac{1}{N}\sum_{i=1}^{N}\left(r\left(x_{i}\right)-x_{i}\right)$, $a_{1}=1.0$, and $a_{n}=0$ for n≥2, respectively. ^ Calibration parameter set is achieved by performing 1st order polynomial regression. ^†^ Calibration parameter set is achieved by performing 3rd order polynomial regression. Note: *CC* is the correlation coefficient of the data pairs and $R^{2}=CC*CC$. *MAE* and *SD* are calculated using Equation (2) and (3), respectively.
